# Sevelamer arsenite nanoparticle as a Pi-responsive drug carrier and embolic agent for chemoembolization

**DOI:** 10.1080/10717544.2022.2072541

**Published:** 2022-05-09

**Authors:** Qiu-Chen Bi, Jian-Jun Tang, Jun Zhao, Yang-Feng Lv, Zhi-Qiang Deng, Hong Chen, Yu-Hua Xu, Chuan-Sheng Xie, Qing-Rong Liang, Rong-Guang Luo, Qun Tang

**Affiliations:** a School of Public Health, Jiangxi Provincial Key Laboratory of Preventive Medicine, Nanchang University, Nanchang, China; b Institute for Advanced Study, Nanchang University, Nanchang, China; c Department of Respiratory and Critical Care Medicine, The First Affiliated Hospital of Nanchang University, Nanchang, China; d Department of Oncology, The First People’s Hospital of Fuzhou, Fuzhou, China; e Department of Interventional Radiology, Jiang Xi Province Chest Hospital, Nanchang, China; f Department of Medical Imaging and Interventional Radiology, The First Affiliated Hospital of Nanchang University, Nanchang, China

**Keywords:** Arsenic trioxide, hepatocellular carcinoma, transarterial chemoembolization, sevelamer, apoptosis

## Abstract

Arsenic trioxide (As_2_O_3_, ATO) has limited therapeutic benefit to treat solid tumors, whether used alone or in combination. Nanoscale drug delivery vehicles have great potential to overcome the limitation of the utility of ATO by rapid renal clearance and dose-limiting toxicity. Polymeric materials ranging from gelatin foam to synthetic polymers such as poly(vinyl alcohol) were developed for vascular embolic or chemoembolic applications. Recently, we have introduced sevelamer, an oral phosphate binder, as a new polymeric embolic for vascular interventional therapy. In this paper, sevelamer arsenite nanoparticle with a polygonal shape and a size of 50–300 nm, synthesized by anionic exchange from sevelamer chloride, was developed as a Pi-responsive bifunctional drug carrier and embolic agent for chemoembolization therapy. At the same arsenic dosage, sevelamer arsenite-induced severer tumor necrosis than ATO on the VX2 cancer model. *In vitro* tests evidenced that Pi deprivation by sevelamer could enhance ATO’s anticancer effect. The results showed that ATO in Pi starvation reduced cell viability, induced more apoptosis, and diminished the mitochondrial membrane potential (Δψm) of cells since Pi starvation helps ATO to further down-regulate Bcl-2 expression, up-regulate Bax expression, enhance the activation of caspase-3 and increase the release of cytochrome c, and the production of excessive reactive oxygen species (ROS). Sevelamer arsenite not only plays a Pi-activated nano-drug delivery system but also integrated anticancer drug with embolic for interventional therapy. Therefore, our results presented a new administration route of ATO as well as an alternative chemoembolization therapy.

## Introduction

Hepatocellular carcinoma (HCC) is the third leading cause of cancer death, and each year over one million new cases was reported all over the world. Despite extensive exploration for novel anticancer drugs and therapeutic strategies, there has achieved little success in improving the treatment of HCC (Asghar & Meyer, 2012; Scudellari, 2014). Surgery is only feasible for very few patients in the early stage (<15%). Massive cytotoxic agents have been developed to treat HCC alone or combined with embolotherapy for over 30 years, but there is a lack of definite evidence that it prolongs survival, including arsenic trioxide (As_2_O_3_; ATO) (Boulin et al., 2011; Ca et al., 2012).

ATO has been approved for the treatment of refractory acute promyelocytic leukemia (APL) since it was first proposed by Chinese clinicians in the 1970s (Shen et al., 1997). Several mechanisms of action have been proposed for As_2_O_3_ activity, including induction of apoptosis mediated by reactive oxygen species (ROS), promotion of cellular differentiation, and inhibition of angiogenesis (Miller et al., 2002; Lu et al., 2007). Pre-clinical studies of As_2_O_3_ have shown antitumor activity in murine solid tumor models (Dilda & Hogg, 2007). Unfortunately, little or no efficacy has been observed in clinical trials of As_2_O_3_ to treat solid tumors (Murgo, 2001). Two factors seem to have limited the utility of As_2_O_3_ in the clinic: rapid renal clearance, which limits tumor uptake, and dose-limiting toxicity (Ahn et al., 2010). Several nano-drug delivery systems (liposome, nanobins, m-SiO_2_, albumin) carrying ATO have great potential to overcome this disadvantage but are still on the way to the bedside (Fu et al., 2020).

Transarterial chemoembolization (TACE) technique, selectively occludes tumor-feeding arteries via transcatheter injection of lipiodol and chemotherapeutic agents, inducing ischemic necrosis of target tumors. It has been confirmed that TACE is the safest, most effective, and best non-surgical method to treat HCC patients in the intermediate stage or to downstage HCC before surgery (Llovet & Bruix, 2003). However, conventional TACE is still faced with high recurrence and low 5-year survival rates. ATO has been combined with embolization therapy to treat HCC with different administrations including transarterial injection with lipiodol (Lv et al., 2017). Recently, we upgraded the conventional embolization technique, where sevelamer chloride particles occlude the tumor-feeding artery, thereby inducing intratumoral inorganic phosphate (Pi) stress and in this Pi-stressed environment, ATO might play a better therapeutic effect on the treatment of HCC (Bi et al., 2022). Sevelamer is a cationic polymer neutralized by chloride or carbonate anions. As a phosphate binder, the protonated amines of sevelamer are ready to bind phosphate anions via both ion exchange and hydrogen bonding interaction to form the insoluble, non-absorbable sevelamer–phosphate complexes, and the complexes are highly stable in the digestive tract (Martin et al., 2011; Yang et al., 2016).

**Scheme 1.**
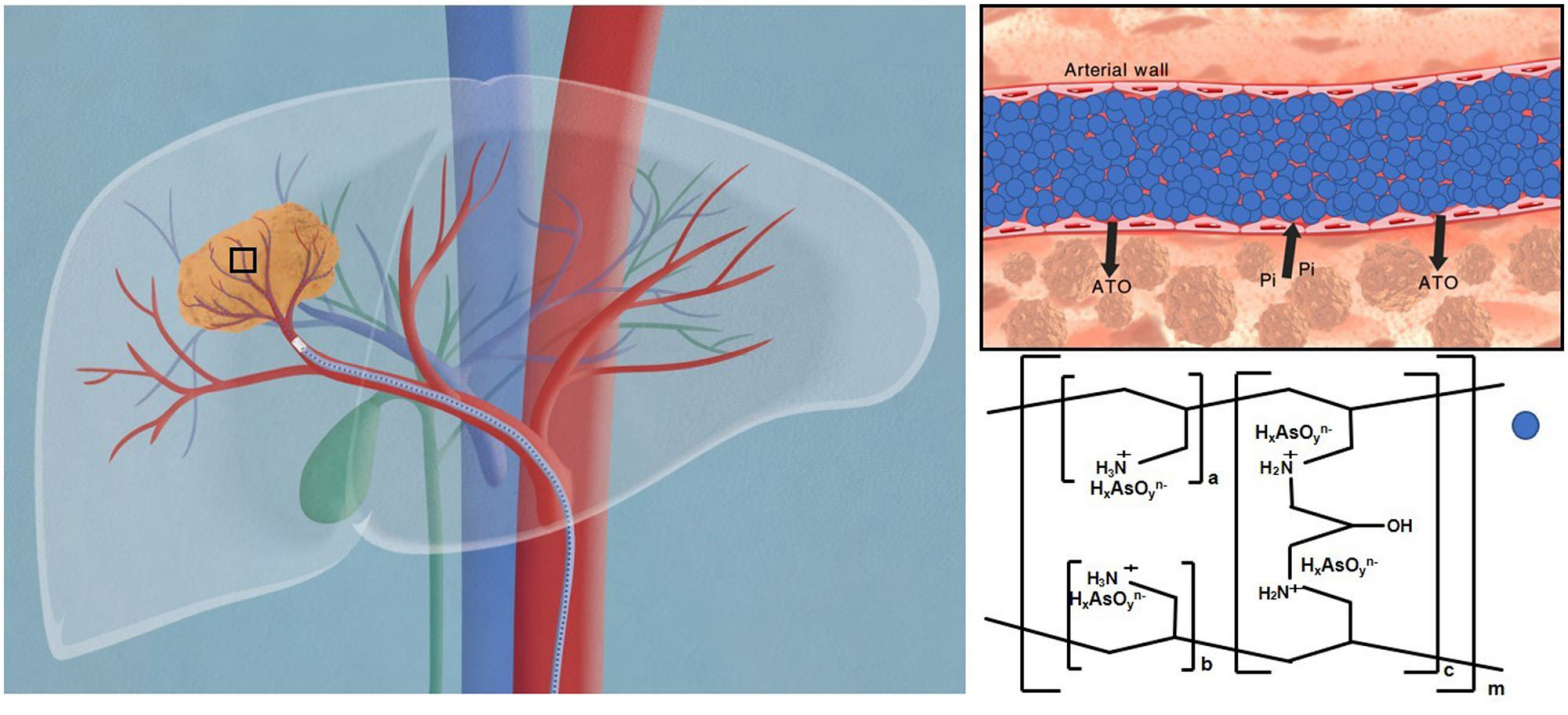
Proposed schematic picture of sevelamer arsenite nanoparticle functioned as ATO delivery and embolic agent under the activation of endogenous inorganic phosphate (Pi) during chemoembolization therapy of HCC. The cartoon on the left described the procedure of transarterial sevelamer arsenite embolization to treat VX2 tumors under the guidance of DSA. The local magnification of the vessel is indicative of Pi-triggered drug downloading and vessel embolization by sevelamer arsenite where arsenite was incorporated into the framework of sevelamer. *In vivo* test evidenced the priority of this new chemoembolization therapy over the conventional TACE therapy. To deeply understand the ATO’s enhanced anticancer effect under low Pi stress, Cell viability, apoptosis, expression of Bcl-2, Bax, caspase-3, and release of cytochrome c into the cytosol, mitochondrial membrane potential (Δψm) as well as intracellular reactive oxygen species (ROS) were examined in the normal and low Pi concentration as ATO was incubated with VX2 cells.

ATO existed in the mixture of anionic arsenite ions (H_2_AsO_3_^–^; HAsO_3_^2–^) under the physiological condition, expecting to substitute chloride or carbonate cations in the sevelamer framework to form a comparably stable sevelamer arsenite as an intermediate product. Sevelamer arsenite was trans-arterially administrated into the tumor-feeding artery via a super-selective interventional technique, where arsenite anions are expected to be replaced by intratumoral phosphate coming from serum, interstitial, or tumor cells, thereby ATO released into the tumor from the sevelamer framework, leaving sevelamer nanoparticle to embolize the vessels, which provides a new strategy for the treatment of HCC (Scheme 1). Different from conventional TACE therapy where ATO was mixed with lipiodol, ATO released from sevelamer arsenite exerts its anticancer effect on the condition of intracellular Pi starvation.

## Materials and methods

### Cell lines and reagents

VX2 cell was maintained in DMEM, supplemented with 10% (v/v) FBS. Sevelamer hydrochloride was purchased from Wuhan Hezhong Pharmaceutical Co., Ltd. (Wuhan, China). Sodium arsenite powder (99%) was bought from Sinopharm (Beijing, China) without purification. The Annexin V-FITC/PI (propidium iodide) apoptosis detection kit was obtained from 4A BIOTECH (Beijing, China). Mitochondrial membrane potential assay kit with JC-1 and ROS Assay Kit were obtained from Beyotime Institute of Biotechnology (Shanghai, China) and One-step TUNEL Apoptosis Kit was provided by Elabscience (Wuhan, China). The antibodies for Bax, Bcl-2, caspase-3, cytochrome c, and β-actin were obtained from Proteintech (Wuhan, China).

### Preparation and characterization of arsenite sevelamer nanoparticle

Arsenite sevelamer nanoparticles were prepared via a co-grinding method. Sodium arsenite and the same molar of sevelamer chloride (0.56 mM) were dissolved in 10 mL deionized water and adjusted to pH 12 with 1 M NaOH. The mixture was put with inert balls (Φ15 mm:Φ5 mm:Φ2 mm:Φ0.5 mm = 50 g:50 g:50 g:50 g) into the autoclave, ground at the speed of 300 RPM for 20 h in 30 mL water in QM-3SP4 planetary ball mill (Nanjing Nanda Instrument Co., Ltd., Nanjing, China), and re-milled (Φ0.05 mm = 200 g) for extra 6 h. The excess unbound ions were removed by centrifugation with deionized water. Arsenite sevelamer nanoparticles were lyophilized for storage. The morphology and the size distribution of the nanoparticle were achieved on scanning electron microscopy (SEM, Quanta 200, Tokyo, Japan) and Malvern Zetasizer Nano ZS90 (Malvern Instruments, Ltd., Worcestershire, UK). To calculate the mass of ATO loading on the sevelamer framework, 0.025 mg arsenite sevelamer nanoparticles were decomposed by heating in the potassium perchlorate–nitric acid mixture and arsenic content were qualified by inductively coupled plasma-MS (ICP-MS).

### Release of ATO from arsenite sevelamer by deprivation of serum Pi

The sevelamer arsenite nanoparticles containing 1 mg arsenic in saline were transferred into a dialysis tube (Spectrum, WMCO 1.5 kDa), and then immersed in a 40 mL beaker filled with plasma ([Pi] = 1.2 mmol/L) at 37 °C. At each designed time point (0, 0.25, 0.5, 1, 2, 12, 24, 48, and 72 h) 0.1 mL of plasma was taken out to quantitatively analyze the arsenic concentration (ICP-MS, Varian 820-MS, Mulgrave, Australia) in the plasma after decomposition in hot concentrated nitric and perchloric acid (9:1), respectively. Pi concentration in the plasma was qualified by the phosphomolybdate method (Beckman Coulter AU2700, Brea, CA). Release curves were plotted against designed time versus plasma Pi or arsenic concentration. All the releasing experiments were repeated three times to alleviate possible errors.

### VX2 animal cancer model

All animal experiments are approved by the Ethics Committee of Nanchang University and implemented in strict accordance with the approval (no. 20190856987). White New Zealand rabbits (3–3.5 kg) were used to graft VX2 liver cancer. In detail, frozen rabbit VX2 tumor tissues were thawed rapidly at 37 °C. The tumor tissue was cut into fragments of around 1 mm^3^ and then the individual was intramuscular injected into the hind limb muscle of the rabbits. Approximately, 2 weeks later, the tumor was harvested and implanted into the left liver tissue via mini-laparotomy. According to our experiments, 15 days later the size of the VX2 tumor will grow up to 1.5–2 cm, suitable for embolization therapy.

### Transcatheter arterial embolization or chemoembolization

As a general embolization protocol, after anesthetization, VX2 rabbits were treated by unilateral femoral puncture with a 4-F vascular sheath (Cook, Bloomington, IN), and a 4-F catheter (Cordis, Miami Lakes, FL) was inserted within this sheath. Then, the 1.98-F catheter (Asahi Intecc, Seto, Japan) was administered into the target artery by super-selective transcatheter technique through a guidewire with 0.018-inch-diameter (Asahi Intecc, Seto, Japan). VX2 rabbits were randomly divided into four groups (*n* = 6), 2 mL lipiodol (first group), 1 mg gridded sevelamer carbonate in 2 mL iodixanol (second group), 0.40 mg ATO in 2 mL iodixanol (third group), and 1 mg sevelamer arsenite in 2 mL iodixanol for the fifth group was carefully delivered via inserted catheter, respectively. After embolization, the catheter was removed and the wound was sutured. For the third and fourth groups, blood was collected from the ear marginal vein at predetermined time points (10 min, 30 min, 1 h, 3 h, 6 h, 12 h, 1 day, 2 days, and 3 days) after the operation. The concentration of As in the circulating blood system was measured by ICP-MS after hot decomposition. Finally, animals are returned to their cages and followed up with daily care until sacrifice. The sixth group (*n* = 6) was randomly divided into two subgroups, in the first subgroup 1 mg sevelamer arsenite in 2 mL iodixanol was transarterially administered, and saline for the second subgroup, all the six VX2 rabbits were sacrificed 2 h after the procedure, and tumors were excised for quantitative Pi analysis by gridding and centrifugation at room temperature.

### Tumor harvest for pathological processing and TUNEL assay

All the rabbits were euthanized by intravenous injection of a lethal amount of pentobarbital sodium (60 mg/kg) on the 7th day after the procedure. Harvested tumors were dissected from the liver and placed into 10% neutral buffered formalin, sectioned through the center of the tumor at the largest diameter 5-µm thickness, and mounted onto glass slides. Slides were stained by using a hematoxylin–eosin stain. Tumor necrosis rate was expressed as the percentage of viable residue tumor area per section, calculated as (whole tumor area-viable tumor area)/whole tumor area.

Apoptosis was detected by one-step TUNEL *in situ* apoptosis detection kit (Elabscience, Wuhan, China). Briefly, the adjacent slice was selected for digestion (100 μL proteinase K, 30 min) at room temperature, and then consequently incubated in 100 μL of TdT equilibration buffer reaction mixture for 20 min at 37 °C, and 50 μL of labeled working solution in a humidified chamber for 60 min at 37 °C. After being treated with DAPI dye solution for 5 min in the dark, the apoptosis of the slices was recorded by fluorescence microscope and quantified via integration of the fluorescent intensity. DAPI staining of the nuclei was indexed for counting the number of the total cells, and the cell with red fluorescence emission was calculated as TUNNEL-positive. Apoptosis rate was performed using the following equation: apoptosis rate (%)=number of TUNEL-positive cells × 100/total number of nuclei.

### CCK-8 assay for evaluation of cytotoxicity exerted by ATO on Pi starvation

The cytotoxicity of the material was evaluated by the CCK-8 assay (Beyotime, Shanghai, China). Approximately, 5 × 10^7^ VX2 cells were incubated against different ATO concentration (0, 5, 10, and 20 μM) in both normal (Pi = 0.906 mM) and Pi-free medium. After 48 h cultivation, each well was added to 10 μL CCK-8 and incubated for two hours in the dark. Then, the absorbance was measured at 450 nm using PerkinElmer's EnSpire Multilabel Plate Reader (Waltham, MA).

### Apoptosis analysis exerted by ATO on Pi starvation

To detect cell apoptosis, VX2 cells (1.5 × 10^5^) were incubated with normal Pi, Pi-free, ATO (2 μM) in Pi, or ATO (2 μM) in a Pi-free medium for 48 h. Note that the three tests described below followed the same condition for cell culture. The cells were harvested by trypsinization without EDTA and washed twice with cold PBS. After the cells were resuspended in 500 μL of binding buffer, 10 μL of stained Annexin-V-FITC and 5 μL of PI (Shanghai 4ABIO, China) were added to the cell suspension, and then the cell incubated at room temperature in the dark for 15 minutes. Finally, the number of apoptosis cells was measured by FACS flow cytometry (BD, Franklin Lakes, NJ).

### JC-1 assay exerted by ATO on Pi starvation

The mitochondrial membrane potential of VX2 cells in the four groups was detected by a mitochondrial membrane potential detection kit (JC-1) (Beyotime, Shanghai, China) to evaluate the early and late apoptosis of cells. When the mitochondrial membrane potential decreased, the fluorescence excited by JC-1 will gradually change from red to green. Therefore, an increase in the ratio of green and red fluorescence represents a decrease in the mitochondria membrane potential. The experimental procedure of the reagent manufacturer's instructions was carried out. 5 × 10^6^ cells were incubated in 0.5 mL JC-1 staining working solution at 37 °C for 20 min. Then, cells were washed three times with JC-1 staining buffer. Finally, the fluorescence intensity was observed under a laser confocal microscope (Olympus BX53; Olympus Corporation, Tokyo, Japan) and a flow cytometer (CYTEK; CYTEK Corporation, Kansas City, MO), respectively.

### Western blot for quantification of apoptosis-related proteins

VX2 cells in the four groups were lysed in RIPA buffer for 10 min and centrifuged at 12,000 rpm for 15 min at 4 °C. Then, the lysate supernatant total protein content was determined by BCA assay. Equal amounts of protein (30 μg) were separated by SDS-PAGE gel electrophoresis and transferred to 0.22 μm PVDF membrane at 200 mA. After blocking with 5% fat-free milk in TBS-T for 2 h, membranes were incubated with primary antibodies (Bax, 1:1500; Bcl-2, 1:1000; Cyto-c, 1:1000; caspase-3, 1:500; β-actin, 1:4000) overnight at 4 °C in TBS-T. The next day, after being washed with TBS-T buffer for 10 min and three times, membranes were incubated with anti-rabbit or anti-mouse secondary antibodies diluted in TBS-T (1:5000) for 1 h. Then, the membrane was washed three times with TBS-T buffer for 10 min each. Finally, the protein bands were visualized under a Tanon-5200 image analyzer (Shanghai, China).

### ROS assay exerted by ATO on Pi starvation

Cellular ROS expression levels were detected using a ROS assay kit (Beyotime, Shanghai, China). The VX2 cells were incubated with the four mediums for 24 hours, and then the cells were collected (3 × 10^6^/mL) and suspended in diluted DCFH-DA (10 μm). After the cells were incubated at 37 °C for 20 minutes, the cells were washed with PBS three times. The fluorescence intensity of intracellular ROS was detected using a flow cytometer (CYTEK; CYTEK Corporation, Kansas City, MO).

### Statistics

Data were analyzed using Prism software (version 6; GraphPad Software, La Jolla, CA). All quantitative data are expressed in the form of mean ± standard deviation. Differences among multiple groups were evaluated by one-way ANOVA, and differences between two groups were assessed by Student’s *t*-test. *p* Values <.05 were considered a statistically significant differences.

## Results

### Development and characterization of sevelamer arsenite nanoparticle

**Figure 1.**
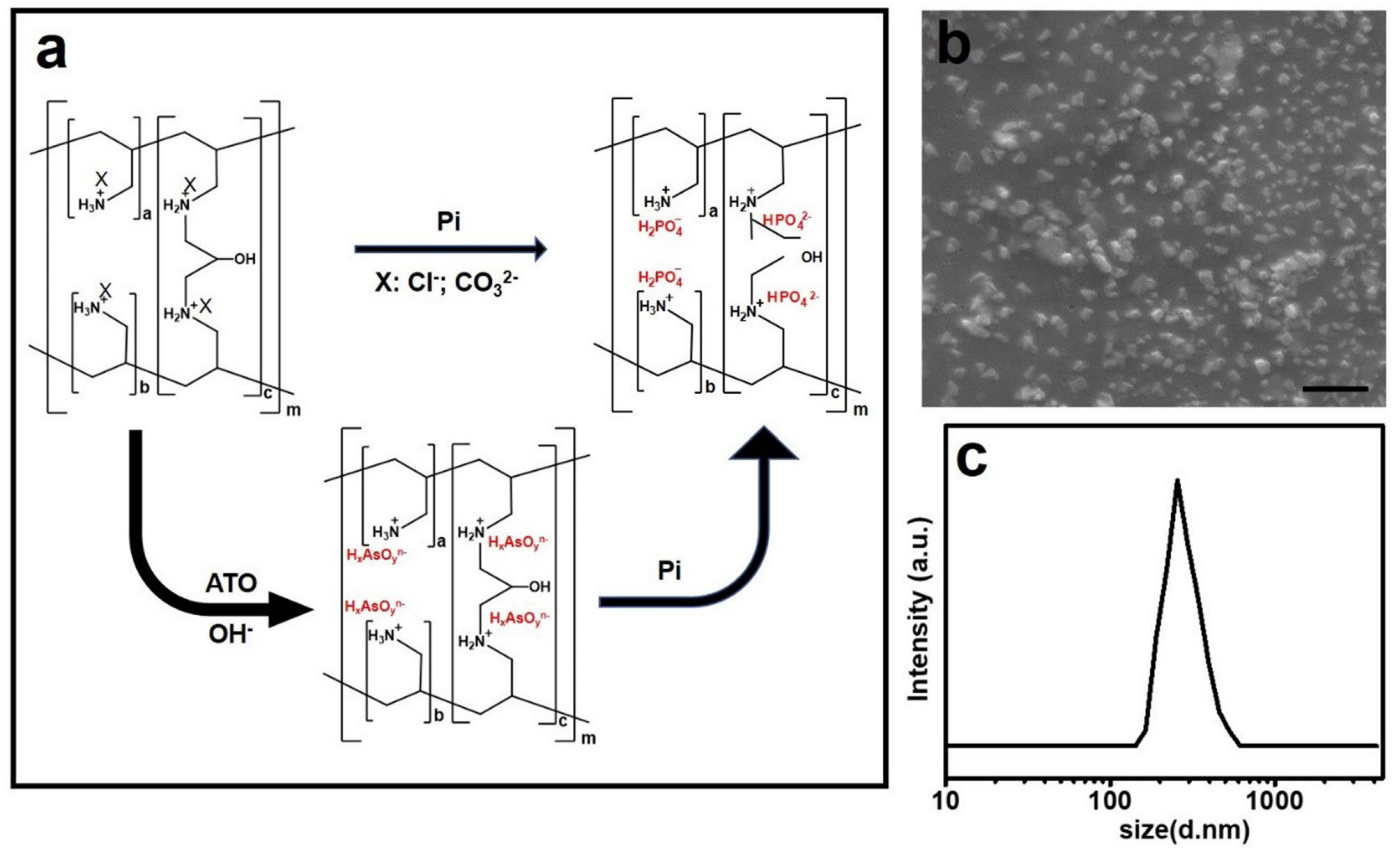
(a) Schematic of uploading and downloading of ATO; (b) SEM images of the sevelamer arsenite nanoparticles, scale bar: 1 µm.

Sevelamer is composed of protonated polyallylamine crosslinked with epichlorohydrin, stabilized by its salt form with carbonate or hydrochloride. The backbone shows a strong binding preference for phosphate over other intestinal anions, such as chlorine and bicarbonate ions due to its dianion character and formation of hydrogen bonding. Arsenite is considered to have a higher binding affinity with the protonated backbone over chloride and bicarbonate, thereby suitable as an intermediate product and ATO carrier (Figure 1(a)). The as-purchased sevelamer chloride has the size of tens of micrometers, after fully gridding with sodium arsenite, chloride was replaced by arsenite anions, as energy-dispersive X-ray (EDX) spectra recorded from individual sevelamer arsenite nanoparticles identified the presence of arsenic (Supplementary Figure S1b), and the sevelamer arsenite is shaped as unregular polygonal particles (Figure 1(b)), consistently with the size and distribution measured by DLS (Figure 1(c)). Note that the surface charge of sevelamer arsenite nanoparticles in Tris–HCl buffer (pH = 7.4) is neutrally charged. The drug loading capacity of 1 g sevelamer is 0.225 g arsenic as determined by ICP-MS.

### Phosphate-triggered ATO release from the sevelamer arsenite nanoparticles

**Figure 2.**
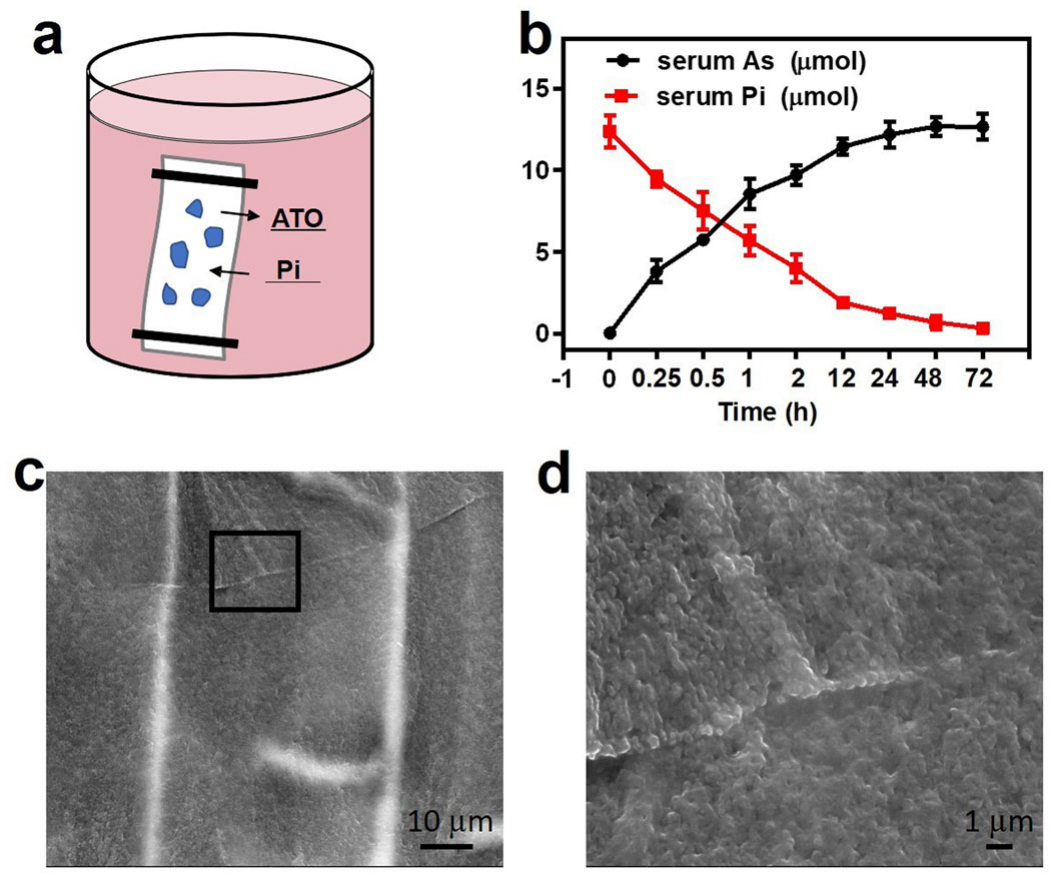
(a) Schematic image of the releasing diagram. (b) Quantitative calculation of arsenic releasing and serum plasma Pi deprivation at designed interval time at 37 °C (*n* = 3). (c) SEM images of the sevelamer–phosphate complex and (d) local magnification of the selected area, are indicative of the formation of dense aggregation.

Anticancer ATO releases upon activation by serum Pi. Pi in the serum is ready to go through the dialysis membrane and react with sevelamer arsenite. The replacement reaction was initiated since sevelamer phosphate is more stable than sevelamer arsenite. The release profiles are shown in Figure 2(a,b). ATO release from sevelamer arsenite nanoparticles and Pi exhaustion in the serum exhibited a time-dependent manner. After ion substitution sevelamer tends to form a densely packed aggregation precipitated in the bottom of the beaker, easy to be detectable by the naked eyes, as evidenced by SEM (Supplementary Figure 2(c,d)) and EDX (Supplementary Figure S1b). This aggregation is very helpful to occlude the tumor-feeding artery in the coming chemoembolization tests.

### Sevelamer arsenite nanoparticle for Pi-deprived chemoembolization therapy

**Figure 3.**
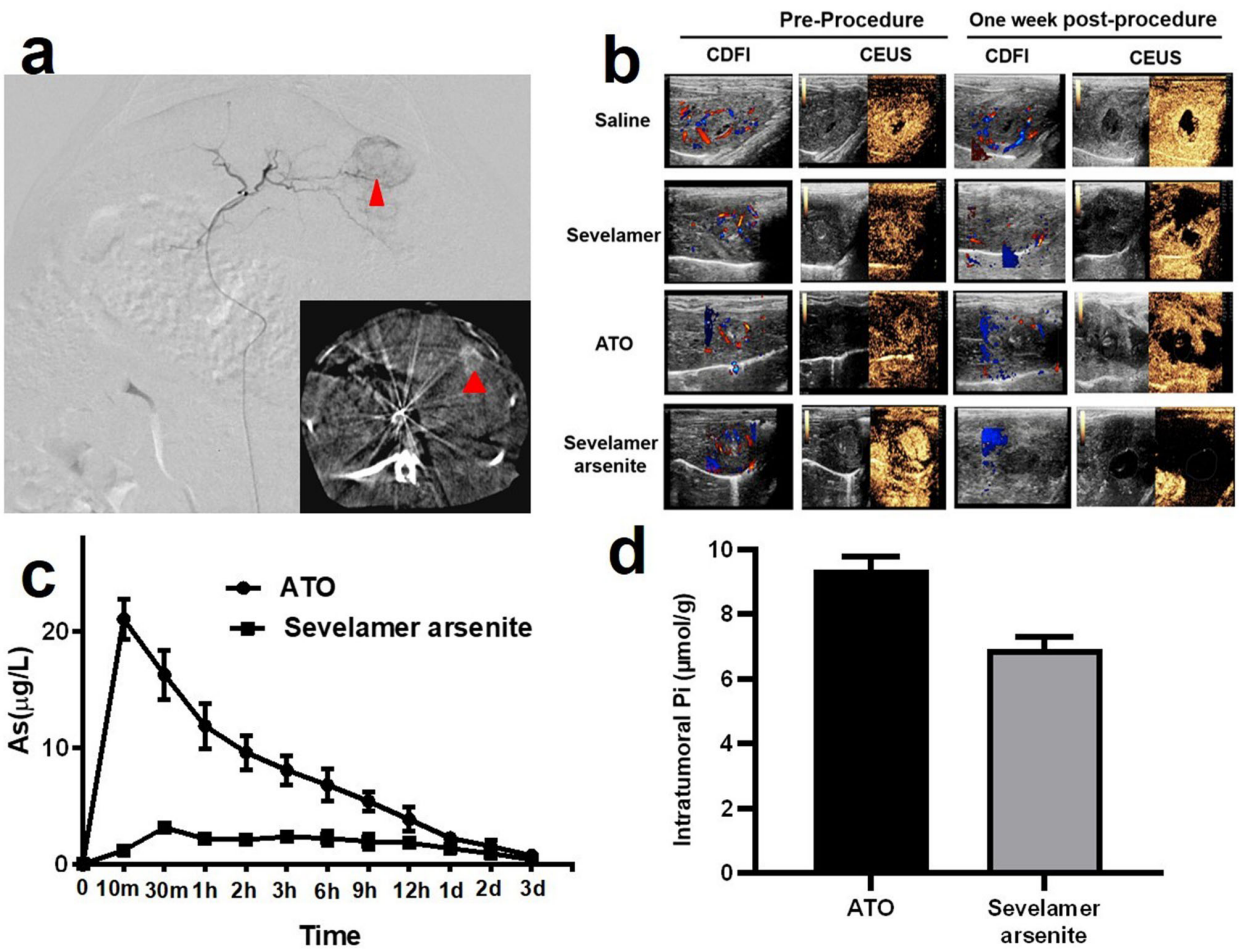
(a) Representative rabbit hepatic DSA images revealed a typical ‘tumor staining (red arrowhead)’, and postembolization serial CT images evidenced of deposited iopamidol (red arrowhead, inserted image). (b) Typical CDFI and CEUS images of VX2 tumors in saline, ATO, sevelamer, and sevelamer arsenite nanoparticles before and 7 days after the procedure. (c) Measurements of plasma arsenic levels at different time points by ICP-MS. (d) Intratumoral Pi concentrations in tumor resected from the ATO and sevelamer arsenite groups.

A total of 30 New Zealand white rabbits were enrolled in the five-cohort study, and in the final three cohorts, the arsenic dosage was the same. Before all the procedures, color Doppler flow imaging (CDFI) and contrast-enhanced ultrasound (CEUS) were utilized to evaluate the blood supply and viability of VX2 tumors. Digital subtraction angiographic (DSA) is indicative of ‘tumor staining’, the characteristic of tumors with a rich blood supply (Figure 3(a)). After confirmation of the tumor’s position, four different injections combined with iopamidol were delivered into the tumor-feeding artery under DSA. All the rabbits in the front four cohorts were re-checked by CDFI and CEUS and then sacrificed one week after the procedure, instead of 2 h for the fifth cohort. CDFI supplies the blood flow signals of the VX2 lesions. Note that different color stands for various blood flow turbulent or laminar toward or away from the scanning plane. All the pre-TACE images are indicative of an abundant blood supply for all the VX2 tumors. In the case of the saline group, abundant blood flow could still be observed. In contrast, in the other three groups, the blood flow remarkably dropped down on a different level. Almost no blood flow within VX2 tumors was visible in the fourth group, indicative of vascular obstruction or severe ischemic necrosis. The lack of arterial phase enhancement from CEUS in the fifth group also showed consistent results. In the second and third groups, viable tumor residue still can be observed. All the ultrasonography features of the VX2 tumors in the four groups are recorded in Figure 3(b).

Plasma arsenic concentration in the periphery blood was recorded in the third and fourth groups. The total amounts of arsenic in the plasma, when administered with sevelamer arsenite lipiodol solution, were low (*C*_max_=3.127 ± 0.511 μg/L) at all time points from 10 min to 3 days. In contrast, the profile in the third group was completely different with a steady peak of arsenic concentration in the plasma, as shown in Figure 3(c). The climax in blood arsenic concentration reaches 10 min (*C*_max_=21.048 ± 1.718 μg/L) after injection, decaying to the baseline after 1 day, seven times higher than sevelamer arsenite. Sevelamer arsenite delayed the ATO release from the tumors very slowly, expecting to alleviate the side effects of systemic exposure to ATO. Intratumoral Pi deprivation was evidenced. Two hours later Pi in the VX2 in the fourth group is lower than that in the third group (9.37 ± 0.41 µmol/g vs. 6.93 ± 0.37 µmol/g), indicating that low Pi stress within the VX2 tumor lasted for at least two hours (Figure 3(d)).

### Sevelamer arsenite mediated chemoembolization induce severe tumor necrosis and apoptosis

**Figure 4.**
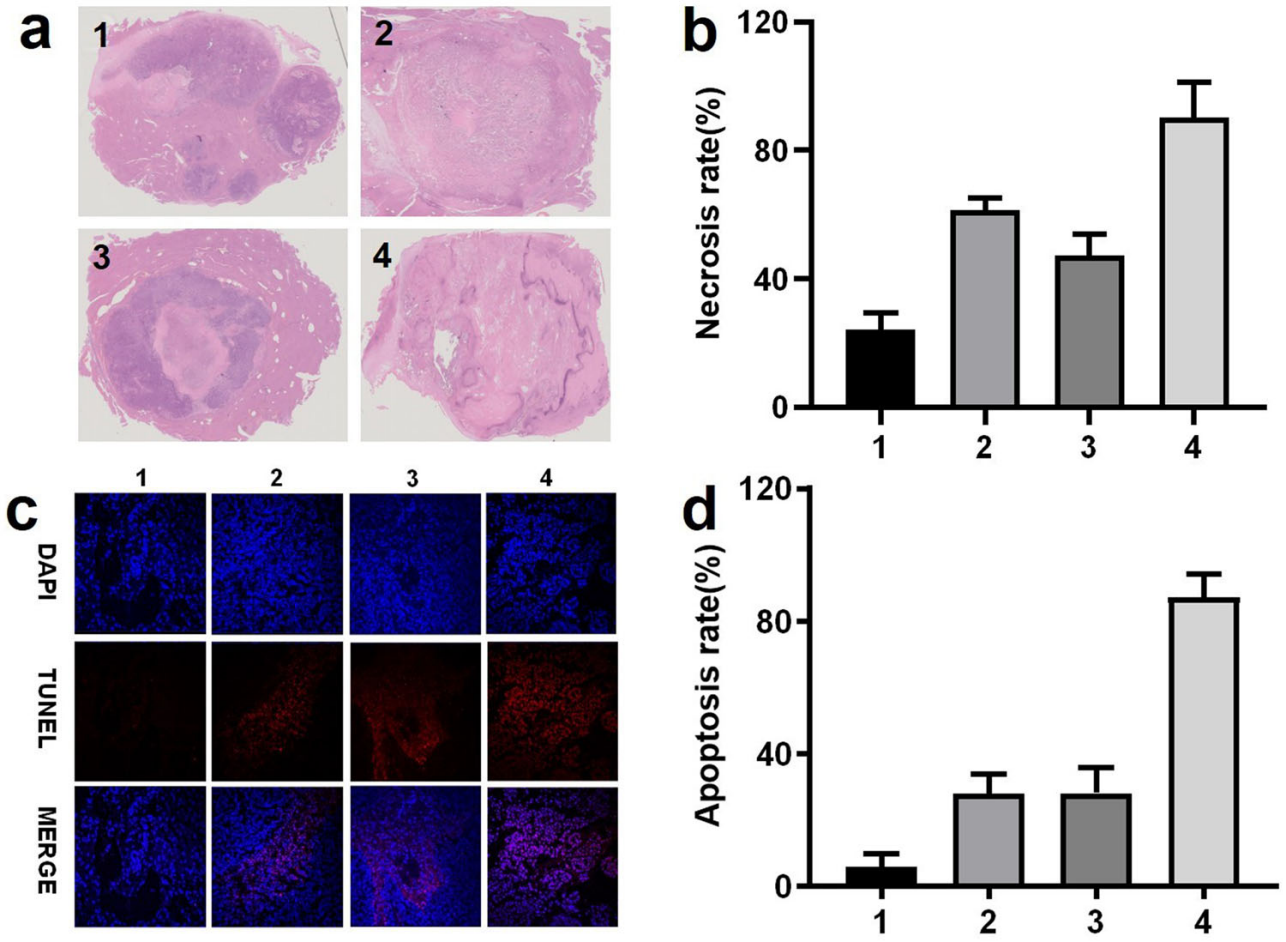
(a, b) Photomicrographs and statistics of the tumor sections staining by H&E (×0.6) treated by saline (group 1), 1 mg ATO (group 2); 2 mg sevelamer (group 3); 2 mg sevelamer arsenite nanoparticle (group 4). (c, d) Representative fluorescent images from TUNNEL staining are indicative of apoptosis (×400, scale bar = 50 μm).

All the rabbits in the fourth cohort were sacrificed at 7 days after the procedure. The tumor necrosis outcomes are displayed in Figure 4(a,b). Administration of either ATO or sevelamer increased the tumor necrosis rate, relative to the sham groups, suggesting that Pi deprivation itself has a moderately therapeutic effect, but not effectively enough to kill all the viable residue (63.5 ± 5.2%). The necrosis ratio in the ATO-TACE group has a similar efficiency (54.6 ± 2.9%). A significant increase in tumor necrosis in the four groups was found (98.65 ± 0.7%). These comparison data indicate that Pi deprivation enhanced the ATO chemotherapy with a synergy effect against HCC.

The representative image recorded by TUNNEL staining and the statistical data are shown in Figure 4(c,d). Note that the region where a large portion of viable tumor residue was selected for identification of the apoptosis but viable cells, recognized by DAPI staining. Administration of ATO and intratumoral in the second and third groups induced a similar apoptosis effect, and complete apoptosis occupied around 30% of all the viable cells. By comparison, administration of sevelamer arsenite nanoparticle-induced apoptosis ratio (number of apoptotic cells/numbers of viable cells) one week after the procedure, indicates that Pi deprivation contributed to the ATO’s apoptosis effect on HCC.

### ATO induced lower viability and higher apoptosis of HCC cells in the Pi-free medium

**Figure 5.**
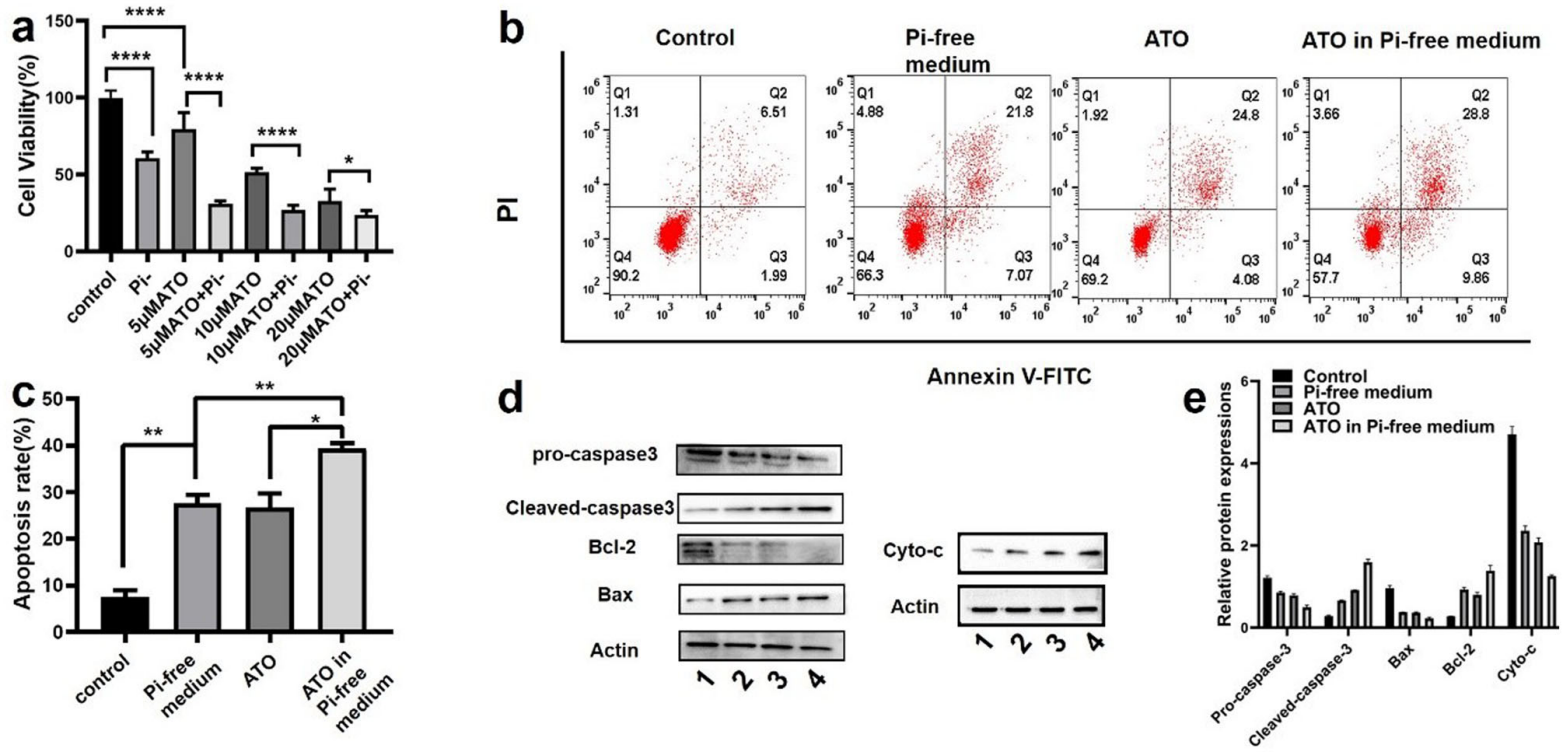
(a) Cell viability of VX2 cells incubated with three incremental doses of ATO (5 µM, 10 µM, and 20 µM) in the presence or absence of Pi for 48 h. *p* Values were obtained by the Kruskal–Wallis test followed by Dunn's post hoc test. (b, c) VX2 cell apoptosis in the groups of control, Pi starvation, 5 µM ATO in the presence or absence of Pi for 48 h. (d, e) Apoptosis-related protein expression under the four stresses for 48 h, 1–4 stands for control, Pi starvation, and 5 µM ATO in the presence or absence of Pi, respectively. *p* Values were obtained by the Kruskal–Wallis test followed by Dunn's post-hoc test. Data are expressed as mean ± SD (*n* = 6). *p<.05, **p<.01, ***p<.001.

The *in vitro* cytotoxicity was evaluated in the VX2 cell line by the CCK-8 assay. VX2 cells were incubated in three types of chemical stress: Pi-free medium, ATO in normal medium, and ATO in the Pi-free medium. As shown in Figure 5(a), for all the three incremental doses of ATO, Pi deprivation enhanced its cytotoxicity on VX2 cells as the dose of ATO is elevated, the synergistic effect becomes weakened. Note that VX2 viability is not sensitive to the Pi stress, while even in the medium containing Pi 31.25 mg/L, a quarter of Pi concentration in the normal medium (125 mg/L), VX2 cell proliferation was not remarkably inhibited (Supplementary Figure S1). The Annexin V-FITC/PI assay was observed by flow cytometry (Figure 5(b,c)) to evaluate VX2 cell apoptosis. The percentage of apoptotic cells significantly increased from 5.89 ± 1.6% in the control group to 29.88 ± 3.4% and 27.13 ± 3.1% by treatment of 5 µM ATO and Pi starvation, respectively. Incubation of cells with 5 µM ATO in a Pi-free medium further induced VX2 apoptosis to an unprecedented high level (39.50 ± 1.9%). Expression of apoptosis-related protein was evaluated by western blot, as shown in Figure 5(d,e). The ratio of Bcl-2/Bax was significantly reduced in VX2 cells treated with Pi or ATO alone as compared to that in the control group and dropped to the minimum value along with the synergized incubation. Meanwhile, the protein level of the cleavages of caspase-3 and cyto-c also showed the same tendency.

### Pi deprivation help ATO to diminish Δψm of VX2 cells

**Figure 6.**
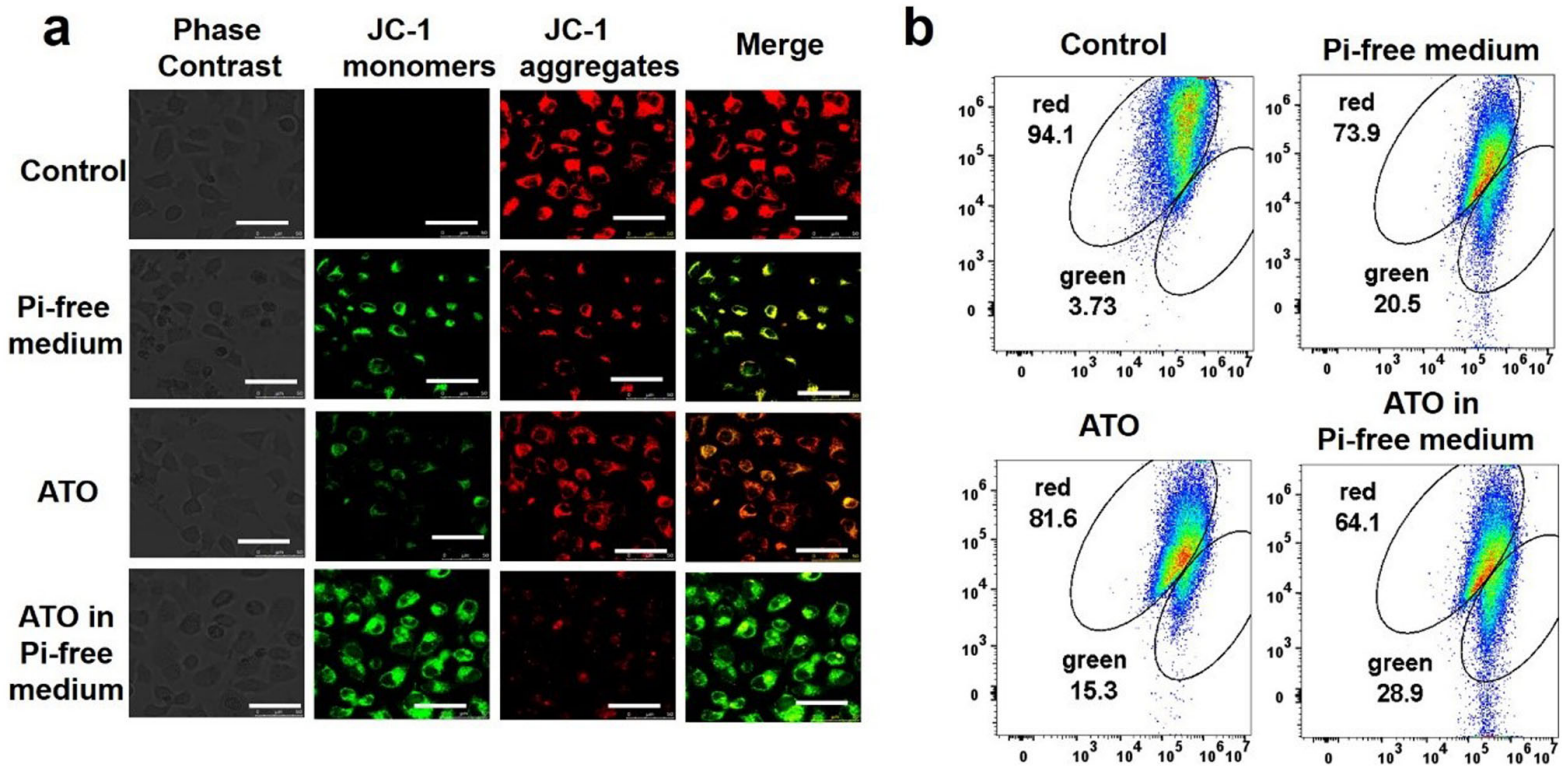
The Δψm change of mitochondria in different groups was recorded by confocal microscopy (a) and flow cytometer (b), scale bar = 50 μm.

The membrane potential of mitochondria (Δψm) of VX2 cells was measured by JC-1 assay. As shown in Figure 6(a,b). The green fluorescence intensity emission from the JC-1 indicator has the same tendency as above. The proportion of green fluorescence cells significantly increased from 3.73% in the control group to 20.5% in the ATO group, 15.3% in the Pi deprivation group, and the maximum value to 28.9% in the ATO-Pi deprivation group, indicating the lowest Δψm value.

### ATO exerts VX2 cells to produce excessive ROS on the condition of Pi deprivation

**Figure 7.**
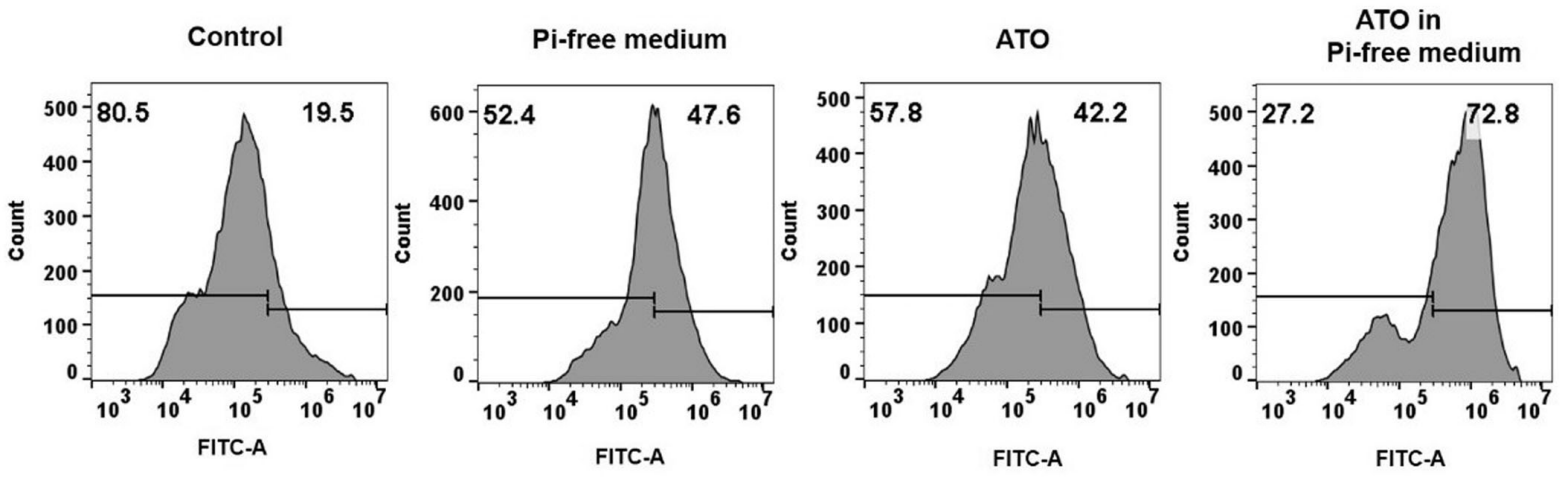
VX2 cells produced different levels of intracellular reactive oxygen species (ROS) in various treated groups.

DCF fluorescence was recorded to quantify the intracellular ROS in the four groups. As shown in Figure 7, the VX2 cells treated with ATO under Pi starvation are ready to produce the highest mass of intracellular ROS, compared to the control group in saline (*p*<.001) and the group treated with only ATO (*p*<.01), as well as Pi starvation alone (*p*<.01).

## Discussion

Sevelamer has a stable framework named as poly(allylamine-co-N,N)-diallyl-1,3-diamino-2-hydroxypropane). As a cationic, insoluble, and polymeric ion exchange resin sevelamer is derived from poly(allylamine hydrochloride) crosslinked with epichlorohydrin in which partial amines are protonated. Sevelamer has been clinically approved as a nonmetallic polymeric phosphate-binding agent. Commercially, chloride or carbonate was utilized to neutralize the positive charge amines by forming a hydrophilic but insoluble powder with the size of tens of micrometers, with a commercial name as Renagel or Renvela, respectively. After oral administration, the protonated amines will dominantly bind phosphate anions via ion exchange and hydrogen bonding to form the non-absorbable sevelamer–phosphate complexes, subsequently eliminated via the feces (Martin et al., 2011; Yang et al., 2016).

Ion exchanged based on the binding affinity between anionic ions drives Pi exhaustion, also accounting for ATO loading and releasing from the sevelamer backbone. Sevelamer arsenite nanoparticle was designed as an ATO carrier, or ATO prodrug, activated by Pi deprivation. After transarterial administration into tumor feeding artery via a super-selective interventional technique, sevelamer arsenite nanoparticles are ready to release ATO, directly into the tumors, and sevelamer swell and aggregate to embolize blood vessels. Both carrying and embolizing processes are activated by endogenous Pi and simultaneously occur. VX2 tumors suffer from three types of stress after this procedure from this single agent: embolization, anti-cancer ATO, and Pi deprivation. Pi starvation promotes the apoptosis of the cells, and leads to a significant increase in the necrosis rate of VX2 tumors, indicating a better therapeutic outcome. *In vitro* test evidenced that Pi starvation help ATO to reduce viability, induce apoptosis, and diminish the Δψm of cells via regulating Bcl-2/Bax expression, enhancing the activation of caspase-3, as well as increasing the release of cytochrome c and the production of intracellular ROS.

ATO has a limited anticancer effect against HCC and is significantly restricted by systemic toxicity (Leu & Mohassel, 2009). Although Nano ATO improves the distribution, metabolism, and excretion pharmacokinetic parameters of drugs *in vivo* to a certain extent, it also faces thorny problems including adverse drug reactions, poor tumor accumulation, and bioavailability (Fu et al., 2020). Moreover, HCC is not sensitive to chemotherapy. How to improve the sensitivity of liver cancer to chemotherapeutic drugs including ATO? The hypoxic environment of tumors is considered to promote drug resistance (Rohwer & Cramer, 2011), meanwhile, recent studies disclose glucose can sensitive HCC cells to doxorubicin and sorafenib (Chouhan et al., 2020). Those findings revealed that changes in the tumor chem-microenvironment may alter the sensitivity of HCC to chemotherapeutic drugs.

In the past 10 years, scientists have discovered that Pi is a new chemo-environmental factor. Some scholars have found that the Pi concentration in the interstitial tumors can be twice times that of normal tissues (Bobko et al., 2017). High concentrations of Pi are associated with tumor progression and metastasis (Sapio & Naviglio, 2015). There is evidence that Pi acts as a signaling molecule in tumor development to activate N-ras to promote cell transformation and skin tumorigenesis (Camalier et al., 2010). Brown & Razzaque summarized that excessive phosphoric acid stress can trigger tumor progression by accelerating angiogenesis, inducing chromosomal instability, and promoting metastasis (Brown & Razzaque, 2018).

Pi deprivation enhances HCC’s sensitivity to anticancer ATO, as we evidenced both *in vivo* and *in vitro* tests. Although the mechanism for the enhancement is complex, Pi-triggered apoptosis has been evidenced. Cellular apoptosis can be triggered by the mitochondrial-apoptosome-mediated intrinsic pathway. Pi deprivation stress-induced mitochondrial transmembrane potential drop-down, resulting in higher membrane permeability and cytochrome c release. The released cytochrome c from mitochondria activates caspase-9, eventually initiating caspase cascade reaction and apoptosis, as rationalized by the theory (Hengartner, 2000; Frion-Herrera et al., 2015). The promoter of apoptosis has been proved to sensitize the killing effect of ATO (Szegezdi et al., 2006; Chen et al., 2017).

Pi starvation enhanced apoptosis of cancer cells via the mitochondrial pathway by additional evidence. Bcl-2 is an anti-apoptotic protein expressed on the surface of the mitochondrial membrane, and this protein prevents changes in Δψm and inhibits the release of cytochrome c. Bax plays the opposite role by binding to Bcl-2 and inhibiting its function. To the greatest extent, ATO inhibits the expression of the Bax and promotes the expression of Bcl-2 by the VX2 cells incubated under Pi-free stress. In all, Pi stress helped ATO promote apoptosis via slowing down the mitochondrial membrane potential of tumor cells and modulating the expression of apoptotic proteins.

The preclinical VX2 animal model tests further evidenced the potential benefit of ATO under Pi starvation. TUNNEL showed that ATO in Pi starvation caused the highest apoptosis rate, consistent with the *in vitro* tests. As the primary response outcome measure in tumor necrosis, associated with survival outcomes after local-regional therapy for HCC (Memon et al., 2011), the enhanced tendency of tumor necrosis is the same as that of apoptosis since apoptotic cells lose their proliferation ability, and eventually die. The increase in apoptosis and necrosis is expected to benefit the treatment of HCC. Note that those enhancement effects were also applied to other human-resourced HCC cells (HEPG-2, SMMC-7721, and MHCC-97H). However, no corresponding animal models are suitable for chemoembolization. This is the major limitation of this study. Additionally, this Pi-stress enhanced effect is not universal for cancer therapy, only for embolization techniques.

## Conclusions

In summary, we designed Pi-responsive ATO-loaded sevelamer nanoparticles for HCC chemoembolization therapy. The as-prepared sevelamer arsenite is ready to exhibit pH-responsive drug release and aggregation. Transarterial sevelamer arsenite has priority over conventional ATO-TACE due to the intratumoral Pi starvation effect. *In vitro* test evidenced that Pi starvation helps ATO to reduce cell viability, induce cell apoptosis, and diminish the mitochondrial membrane potential of cells via regulating Bcl-2/Bax expression, enhancing the activation of caspase-3, as well as increasing the release of cytochrome c and the production of intracellular ROS. This study demonstrated a new nano-formulation of ATO for interventional therapy of HCC.
